# Dose‑dependent association of smoking exposure with HDL subfraction cholesterol content

**DOI:** 10.1038/s41598-026-57573-4

**Published:** 2026-06-11

**Authors:** Péter Pikó, Nóra Kovács, László Pál, Sándor Szücs, Ildikó Seres, György Paragh, Róza Ádány

**Affiliations:** 1 Department of Public Health and Epidemiology, Faculty of Medicine, University of Debrecen, 26. Kassai Street, Debrecen, 4028 Hungary; 2 Epidemiology and Surveillance Centre, Semmelweis University, 25. Üllői Street, Budapest, 1085 Hungary; 3 Department of Public Health and Epidemiology, Faculty of Medicine, HUN-REN Public Health Research Group, University of Debrecen, 26. Kassai Street, Debrecen, 4028 Hungary; 4 Department of Internal Medicine, Faculty of Medicine, University of Debrecen, 98. Nagyerdei Krt, Debrecen, 4032 Hungary; 5 Department of Preventive Medicine and Public Health, Semmelweis University, Budapest, 1085 Hungary

**Keywords:** High-density lipoprotein cholesterol, HDL subfraction cholesterol content, HDL-associated cholesterol distribution, Lipid profile alterations, Regression analysis, Smoking exposure, Risk factors, Molecular medicine

## Abstract

Smoking has a significant effect on plasma lipid concentrations, especially high‑density lipoprotein (HDL)-associated cholesterol and its distribution across HDL subfractions, which are critical for cardiovascular health. The present study aimed to investigate the association between smoking and HDL‑cholesterol (HDL‑C) concentration, as well as the cholesterol distribution between different HDL subfractions, and other lipid parameters, including total cholesterol (TC), low‑density lipoprotein cholesterol (LDL‑C), triglycerides (TG), apolipoprotein AI (ApoAI), apolipoprotein B100 (ApoB100), and the TG/HDL‑C and ApoB100/ApoAI ratios, in a Hungarian cohort of 314 (137 nonsmokers and 177 smokers). HDL‑associated cholesterol was fractionated into ten electrophoretic subfractions (HDL‑1 to HDL‑10) and three subclasses (HDL‑L, HDL‑I, HDL‑S) using the Lipoprint^®^ HDL system. Smoking exposure was characterized using five indicators: current smoking status, cigarettes per day (CPD), duration of smoking (DoS), pack‑years, and the Heaviness of Smoking Index (HSI). Multiple regression models were applied, and significance was defined as *p* < 0.05 after Benjamini–Hochberg correction. All smoking‑related indicators were associated with a decrease in the cholesterol content of HDL subfractions. The strongest associations were observed for HDL‑4, HDL‑5, HDL‑6, and HDL‑7 (*p* < 0.001–0.033), as well as for the HDL‑I subclass (*p* < 0.001). Regarding the broader lipid profile, all indicators were significantly associated with lower HDL‑C (*p* < 0.001) and ApoAI (*p* = 0.001–0.003), and higher TG (*p* = 0.012–0.036), TG/HDL‑C ratio (*p* < 0.001–0.004), and ApoB100/ApoAI ratio (*p* < 0.001–0.015). In conclusion, smoking significantly reduces the cholesterol content of HDL subfractions, particularly within HDL‑4 to HDL‑7 and the HDL‑I subclass. Although total cholesterol levels were not significantly altered, smoking induced an atherogenic shift in lipid composition by lowering HDL‑C and increasing TG levels, which may contribute to or accelerate the development of atherosclerosis.

**Supplementary Information:**

The online version contains supplementary material available at 10.1038/s41598-026-57573-4.

## Introduction

In 2019, 1.14 billion people worldwide were smokers, 7.7 million deaths were directly related to tobacco use, approximately 1.3 million were the result of nonsmokers being exposed to secondhand smoke, and 200 million disability-adjusted life years were attributed to smoking worldwide^1^. Smoking is a major global public health problem, especially in Hungary, as demonstrated by the fact that one in four Hungarian adults report smoking daily, making Hungary the second highest smoking rate in the EU after Bulgaria^2^.

All forms of smoking (cigarette smoking is the most common) are harmful, and there is no safe level of exposure to tobacco. Studies have shown that even low-intensity smoking can significantly increase the risk of premature death^3^ and that smoking intensity, characterized by the number of cigarettes smoked per day (CPD)^4–6^, the duration of smoking (DoS)^7–9^, pack-years^10^ and the Heaviness of Smoking Index (HSI)^11,12^, is strongly related to the severity of relevant health problems. Smoking has a significant effect on the development of cardiovascular diseases (CVDs)^13,14^, which also shows dose-dependent patterns^15^.

The chemicals in tobacco smoke can damage the heart and blood vessels and, through their effects on lipid metabolism, can lead to the build-up of plaque in the arteries, a process known as atherosclerosis^16,17^. Studies have shown that smoking can increase the levels of total cholesterol (TC), triglycerides (TG), and low-density lipoprotein cholesterol (LDL-C) while decreasing high-density lipoprotein cholesterol (HDL-C)^18^. HDL-C helps to remove cholesterol from arteries, so a reduction in its levels due to smoking increases the risk of atherosclerosis^13^ and other heart diseases^19^.

Smoking alters critical enzymes involved in lipid transport and metabolism (such as lecithin-cholesterol acyltransferase – LCAT, cholesterol ester transfer protein – CETP, and hepatic lipase – LIPC)^20^ and the major apolipoproteins (apolipoprotein AI – ApoAI and apolipoprotein B100 – ApoB100)^21^, thereby affecting HDL metabolism and the distribution of cholesterol across HDL subfractions. Smoking-induced oxidative modifications result in HDL dysfunction, which reduces the atheroprotective effect of HDL^22^. Cigarette smoking negatively affects both HDL quantity and its cardiovascular protective function^23^.

HDL is not a single entity but consists of several subfractions, each with unique properties and potential implications for diseases^24^. Beyond their size-based classification, HDL subfractions differ markedly in biological function. Larger, cholesterol‑rich HDL particles contribute predominantly to reverse cholesterol transport, whereas intermediate HDL particles exhibit high ABCA1/ABCG1‑mediated cholesterol efflux capacity^25^ and strong antioxidative and anti‑inflammatory activity^26^. Small HDL particles are more protein‑dense and may reflect enhanced HDL remodeling or catabolism. Because these functional properties vary across subfractions, changes in the cholesterol content of specific HDL subclasses may provide mechanistic insight into how smoking impairs HDL metabolism and cardiovascular protection^27^.

Studies suggest that while larger HDL particles (e.g. HDL_2_) are generally considered protective, smaller HDL (e.g. HDL_3_) may be associated with an increased risk of cardiovascular events^28–31^. Several studies have shown that smoking (both passive^32,33^ and active^22,34^ is associated with a reduction in the HDL_2_ subfraction (especially HDL_2a_^35^, whereas HDL_3_ is only slightly altered by smoking or smoking cessation^22^.

The effect of smoking on the lipid profile is already known from the literature^18,36,37^, but the effects of individual smoking characteristics described by CPD, DoS, pack-years, and HSI on the cholesterol distribution across HDL subfractions, have not been previously investigated. Currently, there is a lack of studies whose results could help to identify more precisely the HDL subfractions altered by smoking, thereby advancing research toward a deeper understanding of the association of smoking with the development of CVD.

The present study aims to investigate the complex relationship between smoking and lipid profiles (TC, HDL-C, LDL-C, TG, ApoAI, and ApoB100 levels, and the TG/HDL-C and ApoB100/ApoAI ratios), as well as the cholesterol content of HDL subfractions determined by the Lipoprint^®^ HDL system. In addition, unlike previous studies in which smoking was generally categorized as never, current, or former smoking, our analyses further examined the association between smoking and lipid parameters using smoking behavior characteristics such as CPD, DoS, pack-years, and HSI.

## Methods

### Study design and populations

A complete and detailed description of the design of the study and the collection of data have been presented in our previous papers^31,38^. Briefly, to understand the background of the poor health status of the Roma population compared with that of the Hungarian population, especially the high incidence of non-communicable diseases, a health survey was designed and implemented to create a complex database for comparative and association statistical analysis. The survey received ethical approval in 2017 (Reference No.: 61327-3/2017/EKU).

The Roma sample is drawn from 25 randomly selected segregated Roma colonies with a population of more than 100. From each colony, 20 households and one person from each household were randomly selected. For the Hungarian general sample, 25 randomly selected individuals from each of the 20 randomly selected general practices of the General Practitioners’ Morbidity Sentinel Stations Program (GPMSSP) were invited to participate in the study.

The study consisted of three main components (a questionnaire-based survey, physical and laboratory examinations) in the Hungarian general (HG) and Roma adult populations aged 20–64 years. A total of 832 participants were included in the survey, 417 from the HG population (232 women and 185 men) and 415 from the Roma population (307 women and 108 men).

The participants provided fasting blood samples for routine laboratory testing (including fasting glucose, glutamic-oxaloacetic transaminase (GOT), TG, HDL-C, LDL-C, and TC measurements) and anthropometric data (such as height and weight), demographic data (such as sex, ethnicity, and age), and socioeconomic and health data (including medication use and blood pressure measurements) were also collected.

Samples were selected from the original population (HG and Roma) according to the following criteria to perform analyses of cholesterol content in HDL subfractions. Participants with missing parameters (20 individuals from the HG and 47 from the Roma) and those receiving lipid-lowering treatment (27 from the HG and 43 from the Roma) were excluded. The remaining 695 subjects (370 from the HG and 325 from the Roma) were divided into two subgroups on the basis of lipid profiles. The normal lipid profile group (126 HG and 87 Roma) included subjects with optimal HDL-C (≥ 1.03 mmol/L in men and ≥ 1.29 mmol/L in women), TG (< 1.7 mmol/L), TC (< 5.2 mmol/L), and LDL-C (< 3.4 mmol/L) levels.

A total of 277 subjects with abnormal lipid profiles (115 from HG and 162 from Roma) and 100 subjects with normal lipid profiles (25 from HG men, 25 from Roma men, 25 from HG women, and 25 from Roma women) were selected to perform analyses of cholesterol content in HDL subfractions.

**Fig. 1:**
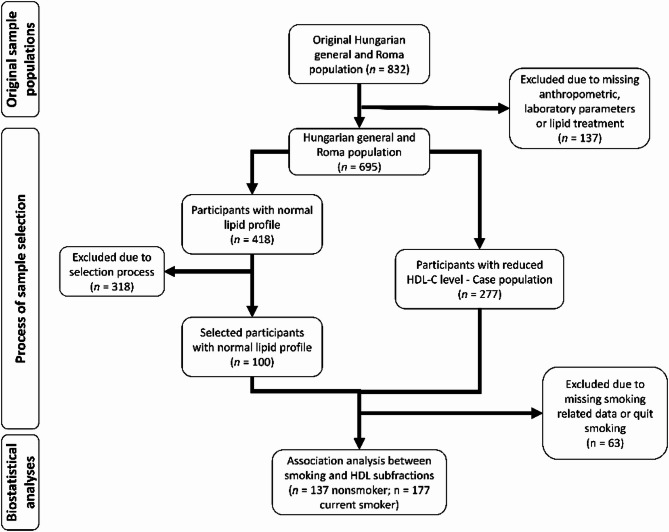
Flowchart showing the process of sample selection.

The subjects of the current study were selected from the HDL subfraction sample population. Those who had quit smoking (22 people) and those with missing data relevant to the present study (*n* = 41) were excluded from further analysis. After these exclusions, 314 subjects (137 nonsmokers and 177 smokers) remained for the current analyses. See Fig. 1 for more details.

### Lipid profile analysis

Lipid levels were determined from fresh serum. TC and TG levels were measured using an enzymatic colorimetric method (GPO-PAP, Modular P-800 Analyzer; Roche/Hitachi, Basel, Switzerland). HDL-C and LDL-C levels were measured using a homogeneous enzymatic colorimetric assay (HDL-C plus 3rd generation, LDL-C plus 2nd generation, Roche, Basel, Switzerland). Apolipoprotein levels were determined by immunoturbidimetric assay Tina-quant Apolipoprotein A-1 (Version 2; Roche, Basel, Switzerland), and Tina-quant Apolipoprotein B (Version 2; Roche, Basel, Switzerland).

Different methods (gel electrophoresis, nuclear magnetic resonance, and ion mobility) are known to separate HDL subfractions^24^, and their unique characteristics determine the number of subfractions that can be separated, e.g., two (HDL_2_ and HDL_3_); three (large, medium, and small HDL), five (HDL_2a_, HDL_2b_, HDL_3a_, HDL_3b_, and HDL_3c_) or ten (from HDL-1 to -10)^39^.

The Lipoprint^®^ HDL system used in the present study separates HDL into 10 electrophoretic bands and quantifies the cholesterol content associated with each band, rather than HDL particle number. These are grouped into three subclasses: large HDL (HDL-L: HDL-1-3), intermediate HDL (HDL-I: HDL-4-7) and small HDL (HDL-S: HDL-8-10). HDL-L corresponds approximately to HDL_2_ (HDL_2a_ and HDL_2b_), as determined by other methods, whereas HDL-I and HDL-S together correspond to HDL_3_ (HDL_3a_, HDL_3b_ and HDL_3c_)^39,40^. Although the system resolves 10 distinct bands, it does not quantify HDL particle number; instead, it measures the cholesterol content associated with each electrophoretically separated HDL subfraction. To our knowledge, only one previous study has examined smoking using this system^41^, and none have evaluated how specific smoking characteristics relate to the distribution of cholesterol across HDL subfractions.

The cholesterol content associated with HDL subfractions was determined using the Lipoprint^®^ HDL Subfractions Testing System (Quantimetrix Corporation, Redondo Beach, CA, USA), which is based on linear polyacrylamide gel electrophoresis. First, 25 µL of serum was added to 3% polyacrylamide gel tubes together with 300 µL of Lipoprint^®^ HDL loading gel solution. The tubes were photopolymerized for 30 min at room temperature using Sudan Black lipophilic dye. Electrophoresis was performed at a constant current of 3 mA/tube for 50 min, alongside the manufacturer’s quality control sample. HDL subfraction cholesterol bands were identified by their relative mobility (rf) and scanned with an ArtixScan M1 digital scanner (Microtek International Inc., Redwood Beach, CA, USA), using very-low-density lipoprotein cholesterol (VLDL-C) + LDL-C as the starting reference (rf 0.0) and albumin as the ending reference (rf 1.0).

Ten HDL‑associated cholesterol bands were distinguished between these reference points and grouped into HDL‑L, HDL‑I and HDL‑S subclasses. The Lipoware software (LW03‑v.16‑134; Quantimetrix Corp., Redondo Beach, CA, USA) calculates the cholesterol content of each HDL subfraction by multiplying the relative area under each band by the total HDL‑C concentration of the sample. Thus, the Lipoprint system quantifies cholesterol content in each HDL subfraction, not HDL particle concentration.

The assay provides both percentage values (relative distribution of cholesterol across subfractions) and concentration‑based values (absolute cholesterol content in mmol/L). In the present study, all analyses were performed using concentration‑based values, as these more accurately reflect quantitative alterations in cholesterol distribution associated with smoking.

The Lipoprint HDL assay includes manufacturer‑provided liquid quality control (QC) material, which was run with each electrophoresis batch. The method is based on a non‑denaturing, linear polyacrylamide gel electrophoresis system that ensures consistent separation of the 10 HDL‑associated cholesterol bands. Published validation studies of the Lipoprint HDL system report intra‑assay coefficients of variation (CVs) of approximately 2–3% and inter‑assay CVs of 3–5% for HDL subfraction cholesterol measurements. All gels in the present study were processed under identical electrophoretic conditions, and QC samples consistently fell within the acceptable ranges defined by the manufacturer, ensuring reproducibility and analytical stability across batches.

### Data used to assess smoking status

Multiple indices were used to determine individual exposure to smoking: current smoking status, CPD, DoS, pack-years (cumulative smoking), and HSI.

For current smoking status, two subpopulations were selected: current smokers and nonsmokers.

The CPD was measured on a self-reported basis and was set to zero for nonsmokers.

The DoS was calculated by subtracting the year of birth from the year of smoking initiation and set to zero for nonsmokers.

A pack-year is the equivalent of smoking one pack of cigarettes per day for one year. One pack was defined as 20 cigarettes. The sample population was divided into three groups based on pack-years, with nonsmokers (zero pack-years), 19 and fewer, and 20 and more pack-years, the cutoff point on the basis of the U.S. Preventive Services Task Force (USPSTF) guidelines^42^.

The Heaviness of Smoking Index was calculated using the two items of the Fagerström Test for Nicotine Dependence^11^: (1) time to first cigarette after waking (TTFC) and (2)number of cigarettes smoked per day (CPD). TTFC was scored as: 0 = ≥ 60 min; 1 = 31–60 min; 2 = 6–30 min; 3 = ≤ 5 min. CPD was scored as: 0 = 1–10 cigarettes/day; 1 = 11–20; 2 = 21–30; 3 = ≥ 31. The HSI score was obtained by summing the two items (range: 0–6), with higher values indicating greater nicotine dependence. Nonsmokers were assigned a score of 0. Based on the total HSI score, nicotine dependence was categorized as low (0–2), moderate^ (3–4)^, or high (5–6).

These smoking exposure indices capture distinct and complementary dimensions of tobacco use. CPD reflects short-term smoking intensity, whereas DoS represents the duration of exposure. Pack-years integrate intensity and duration, providing an estimate of cumulative smoking burden. In contrast, the Heaviness of Smoking Index reflects nicotine dependence. Because these indices represent partially overlapping but biologically distinct aspects of smoking exposure, they were analyzed separately to allow a more nuanced assessment of their relationships with the cholesterol content of HDL subfractions.

### Statistical analyses

Categorical variables were compared using the χ2 test, while comparisons between two subgroups were made using the Mann-Whitney U-test. The Kolmogorov-Smirnov test was used to determine whether the quantitative variables were normally distributed and, if necessary, Templeton’s two-step method^43^ was used to transform the non-normally distributed variables into normal ones. The Jonckheere-Terpstra trend test^44^ was used to identify the statistically significant trend between the ordinal independent variable and the continuous or ordinal dependent variables.

Correlations between continuous variables were assessed via multiple linear regression analyses. For all types of regression analyses, adjustments were made for relevant confounders affecting smoking and HDL-C levels.

Corrections were made for the following covariates:

- Ethnicity: smoking is more prevalent among Roma^45^ and their HDL-C levels have been shown to be lower compared to the Hungarian general population^46^.
- Age: smoking shows an age-related pattern47,48 and HDL-C levels tend to have a positive effect on health and longevity^49^.
- BMI: BMI shows an inverse association with smoking50 as well as with HDL-C levels^50,51^.
- Systolic blood pressure and antihypertensive treatment: systolic blood pressure has a positive association with smoking^52,53^ and an inverse association with HDL-C levels^52–54^.
- GOT levels: plasma GOT levels show no association with smoking, but a significant inverse association can be observed between GOT and HDL-C levels^55^.
- Homeostatic Model Assessment for Insulin Resistance (HOMA-IR): elevated HOMA-IR is positively correlated with smoking^27^ and associated with lower HDL-C levels^56^.
- Self-reported financial status: less favorable financial status is associated with a higher prevalence of smoking^57^ and a higher likelihood of unhealthier lifestyle, and hence lower HDL-C levels^58^.
- Alcohol consumption: alcohol consumption is positively correlated with smoking risk^59^, while moderate alcohol consumption increases HDL-C levels^59,60^.

The Benjamini‒Hochberg method^61^ was used for multiple analyses of the dependent variables (cholesterol content in HDL subfractions) to avoid type I error, and the corrected p-value less than 0.05 was considered significant^62^. All analyses were performed with SPSS software version 29.0 (IBM, Armonk, NY, USA).

## Results

### General characteristics of the sample population

To compare the characteristics of the sample population, a trend analysis was performed for the three subgroups (nonsmokers, 19 or less pack-years, and 20 or more pack-years). A significant increasing trend was observed across groups for smoking severity and age. Similarly, there was a significant increasing trend in the proportion of Roma individuals, in the proportion of individuals with elevated systolic blood pressure (≥ 140 mmHg) and those receiving antihypertensive treatment, and in terms of the proportion of those with more frequent alcohol consumption in association with smoking severity. A decreasing trend was observed between the proportion of people with higher BMIs, the proportion of people with good or average self-reported financial status, and the smoking severity categories.

Significant differences between the two smoker subgroups were observed in DoS (pack-years ≤ 19: 16.95 vs. pack-years ≥ 20: 30.5; *p* < 0.001), CPD (pack-years ≤ 19: 13.76 vs. pack-years ≥ 20: 24.22; *p* < 0.001), HSI (pack-years ≤ 19: 2.56 vs. pack-years ≥ 20: 4.23, *p* < 0.001), and the distribution of time to smoke after waking up (*p* < 0.001). See Table 1 for details.

**Table 1:** General characteristics of the study population.

		Nonsmoker (*n* = 137)	Pack-years ≤ 19 (*n* = 100)	Pack-years ≥ 20 (*n* = 77)	*p*-value
		Average (95% CI)			
Age (years)		40.01 (37.91–42.12)	34.31 (32.15–36.47)	45.43 (43.33–47.53)	< 0.001*^∞^
Duration of smoking (years)		---	16.95 (14.86–19.03)	30.58 (28.48–32.69)	< 0.001*^Δ^
Number of cigarettes smoked per day		---	13.76 (12.26–15.26)	24.22 (22.31–26.13)	< 0.001*^Δ^
Heaviness of Smoking Index		---	2.56 (2.29–2.83)	4.23 (3.99–4.47)	< 0.001*^Δ^
		Prevalence in % (95% CI)			p-value
Time to smoke after waking up	5 or less minutes	---	35.71 (26.75–45.51)	71.43 (60.69–80.89)	< 0.001*^Ω^
	6–30 min	---	38.78 (29.57–48.63)	27.27 (18.29–37.92)	
	More than 31 min	---	25.51 (14.79–40.28)	1.30 (0.14–5.91)	
Roma		35.04 (27.43–43.27)	69.00 (59.49–77.43)	66.23 (55.23–76.05)	< 0.001*^∞^
Women		65.69 (57.48–73.25)	73.00 (63.73–80.96)	66.23 (55.23–76.05)	0.450^∞^
Body mass index (BMI; kg/m^2^)	Underweight (< 18.5)	2.92 (0.99–6.79)	10.00 (5.26–17.01)	7.79 (3.32–15.36)	0.040*^∞^
	Normal weight (18.5–24.9)	26.28 (19.46–34.09)	37.00 (28.02–46.72)	36.36 (26.28–47.45)	
	Overweight (25–29.9)	42.34 (34.29–50.70)	25.00 (17.31–34.12)	29.87 (20.53–40.69)	
	Obese (≥ 30)	28.47 (21.42–36.41)	28.00 (19.92–37.33)	25.97 (17.19–36.52)	
Systolic blood pressure ≥ 140 mmHg		10.95 (6.54–16.98)	8.00 (3.85–14.53)	24.68 (16.10–35.11)	0.003*^∞^
Antihypertension treatment		20.44 (14.34–27.77)	13.00 (7.49–20.62)	28.57 (19.41–39.31)	0.037*^∞^
Glutamic-oxaloacetic transaminase ≥ 40U/L		5.26 (2.38–10.05)	4.08 (1.39–9.41)	6.49 (2.52–13.65)	0.774^∞^
Diabetic status^¥^	Nondiabetic	32.85 (25.40–41.01)	44.00 (34.56–53.78)	45.45 (34.67–56.57)	0.318^∞^
	Prediabetes	29.93 (22.74–37.95)	27.00 (19.04–36.27)	24.68 (16.10–35.11)	
	Diabetes	37.23 (29.47–45.52)	29.00 (20.80–38.40)	29.87 (20.53–40.69)	
Self-declared financial situation	Very good or good	31.58 (22.44–43.64)	23.00 (13.61–36.74)	12.99 (6.88–21.81)	0.007*^∞^
	Adequate	57.14 (48.65–65.32)	62.00 (52.25–71.06)	55.84 (44.71–66.55)	
	Very bad or bad	11.27 (5.90–20.42)	15.00 (7.58–27.22)	31.17 (18.62–48.76)	
Frequency of alcohol consumption	Never	52.55 (44.21–60.79)	49.49 (39.78–59.24)	50.65 (39.63–61.62)	0.006*^∞^
	Once a month or less often	32.85 (25.40–41.01)	44.44 (34.93–54.27)	27.27 (18.29–37.92)	
	2–4 times a month	8.76 (4.88–14.36)	3.03 (0.86–7.87)	10.39 (5.04–18.65)	
	2 or more times a week	5.84 (2.80–10.71)	3.03 (0.86–7.87)	11.68 (4.30–25.53)	

*p < 0.05; ^∞^: Jonckheere-Terpstra trend test; ^Δ^: Mann-Whitney U-test; ^Ω^: χ2 test; ^¥^: diabetic status - nondiabetic (HOMA-IR < 1.82), prediabetes (HOMA-IR: 1.82–3.64), diabetes (HOMA-IR ≥ 3.64).

### General lipid profile associated with smoking

To assess the association between smoking and the overall lipid profile, we examined multiple potential smoking-related indicators, namely, current smoking status, CPD, DoS, pack-years, and HSI. The TC, HDL-C, LDL-C, TG, ApoAI, and ApoB100 levels, and the TG/HDL-C and ApoB100/ApoAI ratios were used to characterize the general lipid profile.

HDL-C, TG, and ApoAI levels, as well as the TG/HDL-C and ApoB100/ApoAI ratios, were significantly associated with all smoking-related indicators. ApoB100 levels were only significantly associated with DoS, whereas TC and LDL-C levels were not significantly associated with any of the outcome variables. See Table 2 for more details.

**Table 2:** Association of smoking-related indicators with serum lipid concentrations.

	Current smoker		Number of cigarettes smoked per day		Duration of smoking		Pack-years		Heaviness of Smoking Index	
	B (95% CI)	*p*-value	B (95% CI)	*p*-value	B (95% CI)	*p*-value	B (95% CI)	*p*-value	B (95% CI)	*p*-value
HDL-C (mmol/L)	-0.138 (-0.210 – -0.067)	< 0.001*	-0.007 (-0.010 – -0.003)	< 0.001*	-0.005 (-0.008 – -0.002)	< 0.001*	-0.005 (-0.007 - -0.002)	< 0.001*	-0.039 (-0.059 – -0.018)	< 0.001*
TC (mmol/L)	0.000 (-0.224–0.223)	0.999	-0.001 (-0.012–0.010)	0.902	0.003 (-0.006–0.013)	0.480	0.002 (-0.007–0.010)	0.708	-0.010 (-0.075–0.055)	0.760
TG (mmol/L)	0.272 (0.059–0.486)	0.012*	0.012 (0.002–0.023)	0.021*	0.010 (0.001–0.019)	0.026*	0.011 (0.003–0.018)	0.008*	0.066 (0.004–0.128)	0.036*
LDL-C (mmol/L)	0.063 (-0.142–0.268)	0.545	0.001 (-0.009–0.011)	0.784	0.004 (-0.004–0.013)	0.331	0.002 (-0.005–0.010)	0.586	0.002 (-0.057–0.062)	0.940
TG/HDL-C ratio	1.062 (0.465–1.658)	< 0.001*	0.047 (0.018–0.077)	0.002*	0.039 (0.014–0.064)	0.003*	0.038 (0.016–0.059)	< 0.001*	0.260 (0.086–0.433)	0.004*
ApoAI (g/L)	-0.100 (-0.155 – -0.045)	< 0.001*	-0.005 (-0.008 – -0.002)	< 0.001*	-0.003 (-0.006 – -0.001)	0.003*	-0.003 (-0.005 - -0.001)	0.002*	-0.029 (-0.045 – -0.013)	< 0.001*
ApoB100 (g/L)	0.055 (-0.009–0.119)	0.089	0.002 (-0.001–0.005)	0.209	0.003 (0.001–0.006)	0.014*	0.002 (0.000–0.005)	0.050	0.011 (-0.008–0.029)	0.261
ApoB100/ApoAI	0.087 (0.031–0.144)	0.003*	0.004 (0.001–0.006)	0.011*	0.004 (0.002–0.006)	< 0.001*	0.003 (0.001–0.005)	0.003*	0.020 (0.004–0.037)	0.015*

*p < 0.05. TC: total cholesterol, TG: triglycerides, LDL-C: low-density lipoprotein cholesterol, HDL-C: high-density lipoprotein cholesterol, ApoAI: apolipoprotein AI, ApoB100: apolipoprotein B100. Multiple linear regression was adjusted for ethnicity, sex, age, BMI, systolic blood pressure, antihypertensive treatment, glutamic-oxaloacetic transaminase level, diabetic status, self-reported financial status, and alcohol consumption.

### Comparison of HDL-associated cholesterol content between current smokers and nonsmokers

**Fig. 2:**
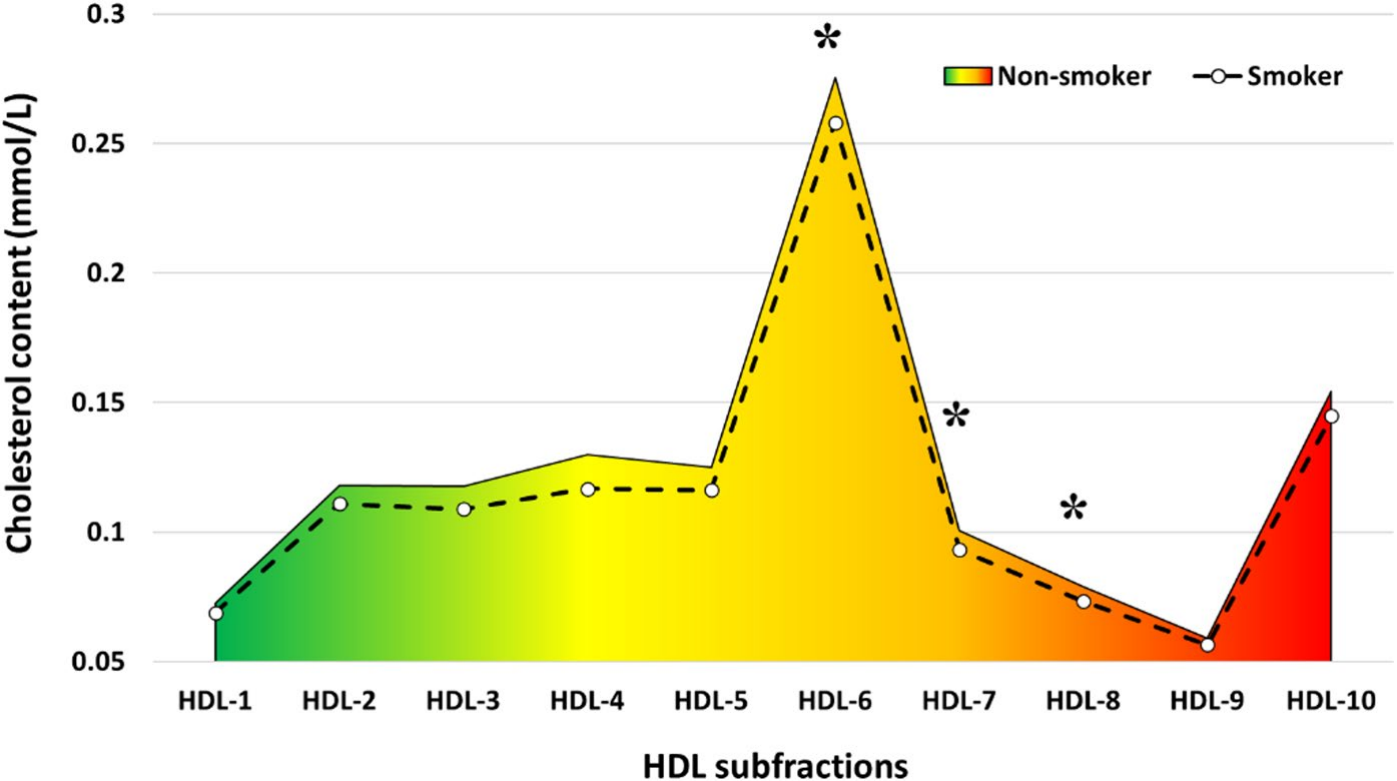
Comparison of the mean cholesterol content of HDL subfractions between current smokers and nonsmokers. Asterisks (*) indicate HDL subfractions with statistically significant differences after Benjamini–Hochberg correction (*p* < 0.05).

Among the ten HDL subfractions tested, the cholesterol content of HDL-6 (nonsmokers: 0.28, 95% CI: 0.26–0.29 vs. smokers: 0.26, 95% CI: 0.25–0.27; *p* = 0.040), HDL-7 (nonsmokers: 0.10, 95% CI: 0.10–0.10 vs. smokers: 0.09, 95% CI: 0.09–0.10; *p* = 0.030), and HDL-8 (nonsmokers: 0.08, 95% CI: 0.08–0.08 vs. smokers: 0.07, 95% CI: 0.07–0.08; *p* = 0.043) was significantly lower in smokers than in nonsmokers. See Fig. 2 for more details.

After Benjamini–Hochberg correction, the cholesterol content of the HDL subclasses did not differ significantly between smokers and nonsmokers, either in absolute concentration (in mmol/L; HDL-L_smoker_: 0.29 vs. HDL-L_nonsmoker_: 0.31, *p* = 0.859; HDL-I_smoker_: 0.58 vs. HDL-I_nonsmoker_: 0.63, *p* = 0.074; HDL-S_smoker_: 0.27 vs. HDL-S_nonsmoker_: 0.29, *p* = 0.260) or in relative distribution (in %; HDL-L_smoker_: 24.20 vs. HDL-L_nonsmoker_: 23.56, *p* = 0.936; HDL-I_smoker_: 51.11 vs. HDL-I_nonsmoker_: 51.46, *p* = 0.654; HDL-S_smoker_: 24.70 vs. HDL-S_nonsmoker_: 24.98, *p* = 0.639).

### Association of smoking-related indices with the cholesterol content of HDL subfractions

The cholesterol content of HDL‑2 to HDL‑8 subfractions and all HDL subclasses (expressed in mmol/L) showed a significant negative association with current smoking status. The strongest association was observed for the cholesterol content of the HDL-I (B = -0.074, 95% CI: -0.110 – -0.038, *p* = 1.65 × 10^− 4^), followed by HDL-6 cholesterol content (B = -0.027, 95% CI: -0.040 – -0.013, *p* = 1.02 × 10^− 3^). See Table 3 for details.

The cholesterol content of HDL‑3 to HDL‑9 and all HDL subclasses was significantly negatively associated with CPD. Again, the strongest associations were observed for HDL‑I cholesterol content (B = -0.004, 95% CI: -0.005 – -0.002, *p* = 4.70 × 10^− 4^) and HDL‑6 cholesterol content (B = -0.001, 95% CI: -0.002–0.000, *p* = 1.41 × 10^− 4^). See Table 3 for more details.

The cholesterol content of HDL‑4, HDL‑5, HDL‑6, and HDL‑7, as well as that of the HDL‑I subclass (B = -0.003, 95% CI: -0.004–0.001, *p* = 1.29 × 10^− 3^), was significantly associated with DoS. Among individual subfractions, HDL‑6 cholesterol content showed the strongest association (B = -0.001, 95% CI: -0.002 – -0.000, *p* = 3.37 × 10^− 3^). See Table 3 for more details.

Pack‑years showed significant negative associations with the cholesterol content of HDL‑4 through HDL‑9, as well as with HDL‑I and HDL‑S subclasses. The strongest associations were again observed for HDL‑6 cholesterol content (B = -0.001 95% CI: -0.001 – -0.001, *p* = 5.88 × 10^− 4^) and HDL‑I cholesterol content (B = -0.003, 95% CI: -0.004–0.001, *p* = 3.78 × 10^− 4^).

The cholesterol content of HDL‑3 through HDL‑10, as well as that of HDL‑I and HDL‑S subclasses, was significantly associated with the HSI. The strongest associations were observed for HDL‑I cholesterol content (B = -0.020, 95% CI: -0.031 – -0.010, *p* = 3.60 × 10^− 4^) and HDL‑6 cholesterol content (B = -0.008, 95% CI: -0.012 – -0.004, *p* = 5.70 × 10^− 4^). See Table 3 for more details.

The relative distribution (%) of cholesterol across HDL subfractions and subclasses was not significantly associated with current smoking status or any smoking‑related indices. See Supplementary Table 1 for details.

**Table 3:** Association of smoking-related indices with the cholesterol content of HDL subfractions.

	Current smoker		Daily number of cigarettes smoked		Duration of smoking		Pack-years		Heaviness of Smoking Index	
	B (95% CI)	*p*-value	B (95% CI)	*p*-value	B (95% CI)	*p*-value	B (95% CI)	*p*-value	B (95% CI)	*p*-value
HDL-1 (mmol/L)	-0.008 (-0.016–0.001)	0.108	-0.000 (-0.001–0.000)	0.645	-0.000 (-0.001–0.000)	0.198	-0.000 (-0.000–0.000)	0.460	-0.001 (-0.003–0.002)	0.547
HDL-2 (mmol/L)	-0.014 (-0.026 – -0.001)	0.049*	-0.001 (-0.001–0.000)	0.106	-0.001 (-0.001–0.000)	0.082	-0.000 (-0.001–0.000)	0.157	-0.003 (-0.006–0.001)	0.181
HDL-3 (mmol/L)	-0.020 (-0.034 – -0.005)	0.014*	-0.001 (-0.002–0.000)	0.018*	-0.001 (-0.001–0.000)	0.055	-0.001 (-0.001 – -0.000)	0.056	-0.005 (-0.009 – -0.001)	0.031*
HDL-4 (mmol/L)	-0.021 (-0.034 – -0.008)	0.005*	-0.001 (-0.002–0.000)	0.010*	-0.001 (-0.001 – -0.000)	0.026*	-0.001 (-0.001 – -0.000)	0.033*	-0.005 (-0.009 – -0.001)	0.024*
HDL-5 (mmol/L)	-0.014 (-0.022 – -0.006)	0.003*	-0.001 (-0.001–0.000)	0.003*	-0.001 (-0.001 – -0.000)	0.010*	-0.000 (-0.001 – -0.000)	0.005*	-0.004 (-0.006 – -0.001)	0.007*
HDL-6 (mmol/L)	-0.027 (-0.040 – -0.013)	< 0.001*	-0.001 (-0.002–0.000)	< 0.001*	-0.001 (-0.002 – -0.000)	0.003*	-0.001 (-0.001 – -0.001)	0.001*	-0.008 (-0.012 – -0.004)	0.001*
HDL-7 (mmol/L)	-0.010 (-0.016 – -0.004)	0.002*	-0.000 (-0.001–0.000)	0.002*	-0.000 (-0.001 – -0.000)	0.012*	-0.000 (-0.001–0.001)	0.004*	-0.003 (-0.005 – -0.001)	0.002*
HDL-8 (mmol/L)	-0.006 (-0.011 – -0.001)	0.021*	-0.000 (-0.001–0.000)	0.029*	-0.000 (-0.000–0.000)	0.076	-0.000 (-0.000 – -0.000)	0.038*	-0.002 (-0.003 – -0.000)	0.024*
HDL-9 (mmol/L)	-0.003 (-0.007 – -0.001)	0.099	-0.000 (-0.000–0.000)	0.032*	-0.000 (-0.000–0.000)	0.209	-0.000 (-0.000 – -0.000)	0.047*	-0.001 (-0.003 – -0.000)	0.024*
HDL-10 (mmol/L)	-0.011 (-0.023–0.002)	0.099	-0.001 (-0.001–0.000)	0.065	-0.000 (-0.001–0.000)	0.174	-0.000 (-0.001 – -0.000)	0.058	-0.004 (-0.008 – -0.001)	0.028*
Large HDL (mmol/L)	-0.042 (-0.075 – -0.009)	0.018*	-0.002 (-0.003–0.000)	0.048*	-0.001 (-0.003–0.000)	0.059	-0.001 (-0.002–0.000)	0.084	-0.008 (-0.018–0.001)	0.083
Intermediate HDL (mmol/L)	-0.074 (-0.110 – -0.038)	< 0.001*	-0.004 (-0.005 – -0.002)	< 0.001*	-0.003 (-0.004 – -0.001)	0.001*	-0.003 (-0.004 - -0.001)	< 0.001*	-0.020 (-0.031 – -0.010)	< 0.001*
Small HDL (mmol/L)	-0.021 (-0.040 – -0.002)	0.030*	-0.001 (-0.002 – -0.000)	0.017*	-0.001 (-0.002 – -0.000)	0.064	-0.001 (-0.002 - -0.000)	0.017*	-0.008 (-0.013 – -0.002)	0.009*

*Significant results according to p-values adjusted by the Benjamini‒Hochberg method. Multiple linear regression was adjusted for ethnicity, sex, age, BMI, systolic blood pressure, antihypertensive treatment, glutamic-oxaloacetic transaminase level, diabetic status, self-reported financial status, and alcohol consumption.

## Discussion

The aim of our analysis was to investigate the impact of smoking on the lipid profile, particularly the cholesterol content of HDL subfractions in 137 nonsmokers and 177 smokers. Smoking exposure was measured on the basis of current smoking status, CPD, DoS, pack-years, and HSI.

In the present study, smoking and smoking exposure were significantly negatively associated with HDL-C and ApoAI levels, and positively associated with TG levels, the TG/HDL-C ratio, and the ApoB100/ApoAI ratio. These results are fully consistent with previously published findings showing that smoking adversely alters the lipid profile and accelerates the development of atherosclerosis^18,63–65^.

Our results revealed that smoking significantly reduced the cholesterol content of all HDL subclasses and of the HDL‑2 to HDL‑8 subfractions, but did not affect their percentage distribution. Zhao et al.^41^ reported significantly lower HDL‑L and HDL‑I concentrations (mg/dL) and proportions (%), whereas both the concentration and proportion of HDL‑S were significantly higher in smokers compared with nonsmokers. The findings for HDL‑L and HDL‑I are consistent with our results. In contrast to Zhao et al., who reported higher HDL‑S concentrations and proportions in smokers, in our study smoking was significantly negatively associated with the cholesterol content of HDL‑S after correction, while the percentage distribution of HDL‑S remained unchanged. This apparent discrepancy likely reflects methodological differences: Zhao et al. analyzed subclass proportions and mass concentrations, whereas the Lipoprint system quantifies the cholesterol content within each subfraction.

The difference in results may also be influenced by sample characteristics. Zhao et al. used a larger sample size (nonsmokers: *n* = 518 vs. *n* = 137; smokers: *n* = 256 vs. *n* = 177), but their groups differed markedly in sex composition (proportion of men: 41.3% among nonsmokers vs. 93.0% among current smokers), which strongly affects HDL levels and subfraction profiles. Additional factors such as lifestyle, environmental exposures, or genetic background may also contribute. Nevertheless, other studies using different HDL separation methods have similarly shown that smoking predominantly reduces the HDL_2_ subfraction (corresponding to HDL‑L), whereas HDL_3_ (corresponding to HDL‑I and HDL‑S) is only slightly altered^35,66,67^. Comparable findings have been reported in passive smokers, who exhibit significantly lower HDL_2_ levels than nonsmokers^68^.

A significant negative correlation with the CPD was observed for total HDL-C, and the HDL-3 to HDL-9 subfractions and for all three subclasses. In addition, total HDL‑C, the cholesterol content of HDL‑4 to HDL‑7 subfractions, and the cholesterol content of the HDL‑I subclass were negatively correlated with DoS, consistent with previous studies in the literature^13,63,69^. However, the associations of CPD and DoS with the cholesterol content across HDL subfractions have not been previously investigated.

Pack‑years were significantly negatively associated with the cholesterol content of HDL‑4 to HDL‑9, as well as with HDL‑I and HDL‑S. According to previous studies, pack-years were significantly associated with lower HDL-C levels^63^, and showed a weak correlation with the monocyte-to-HDL-C ratio^70^. The associations of pack-years with the cholesterol content across HDL subfractions have not been previously investigated.

HSI was significantly associated with lower cholesterol content in the HDL‑I and HDL‑S subclasses and the HDL-3 to HDL-10 subfractions. HSI is a tool used to measure the intensity of a person’s smoking habit and potential nicotine dependence. Nicotine levels are negatively correlated with HDL-C levels^71,72^. Previous studies have not investigated the associations of the HSI with HDL-C and its subfraction profile.

The partly different association patterns observed for CPD, DoS, pack-years, and HSI suggest that smoking intensity, duration, cumulative exposure, and nicotine dependence may influence HDL metabolism through overlapping but not identical pathways.

The smoking-related reductions in HDL-associated cholesterol across subfractions observed in the present study have important functional implications. In particular, the HDL-I subclass (HDL-4 to HDL-7), which showed the most consistent decreases across smoking indicators, has been linked to metabolically active HDL functions with a prominent role in reverse cholesterol transport and in ABCA1/ABCG1-mediated cholesterol efflux^73^. A reduction in HDL-associated cholesterol in these subclasses may therefore indicate impaired HDL remodeling and a diminished capacity for cholesterol efflux^74^, which can be accompanied by reduced antioxidative and anti-inflammatory properties^26^. These findings are consistent with prior work showing that cigarette smoking impairs HDL quantity and function, alters lipid transfer processes and enzyme activity, and promotes oxidative modification of ApoAI, thereby contributing to the formation of smaller, less functional HDL particles^22^. Clinically, such changes may represent a shift toward a more atherogenic HDL phenotype and may help explain why smoking is associated with increased cardiovascular risk^75^.

Based on the results of our previous studies, the cholesterol content of HDL‑1‑6 subfractions and HDL-L and HDL-I classes were significantly negatively associated with the progression and persistence of insulin resistance^56^ and the cholesterol content of HDL-1-3 subfractions and HDL-L levels were significantly inversely associated with estimated cardiovascular risk^31^.

The development of CVD, particularly the process of atherosclerosis, is closely related to the quantitative and qualitative characteristics of HDL^26,76,77^. Recent evidence indicates that the cholesterol content across HDL subfractions may be a better marker for predicting cardiovascular events than total HDL-C levels^78^. The size and composition of HDL particles are heterogeneous due to the modification of the associated enzymes (CETP and LCAT) by phospholipid transfer proteins^79^. These modifications can lead to changes in HDL function and its conversion to proatherogenic^80^. Small HDL particles are the most effective at removing cholesterol from cells^81^, thus preventing the development of atherosclerosis and other types of CVD^82^.

Among studies using the Lipoprint^®^ HDL Subfractions System, several findings support the heterogeneity and clinical relevance of HDL subfractions. Zitnanova et al. reported that the smallest HDL particles (HDL‑8 to HDL‑10) were significantly higher in men than in women in an atherosclerotic cohort, highlighting sex‑related differences in HDL remodeling^83^. Zhang et al. demonstrated that reduced HDL‑L levels were strongly associated with very early and early coronary artery disease in an untreated Chinese population, underscoring the anti‑atherogenic role of large HDL particles^84^. Chruściel et al. found that HDL‑3 and HDL‑I levels correlated with systolic blood pressure in young adults, suggesting that intermediate HDL particles may reflect early vascular alterations^81^. Furthermore, Otrante et al. showed that cholesterol efflux capacity was positively correlated with HDL‑L levels (*r* = 0.35; *p* < 0.001), indicating that larger HDL particles retain superior functional properties^85^. Together, these studies emphasize that cholesterol content of individual HDL subfractions (particularly HDL‑L and HDL‑I) carry distinct biological and clinical significance, which is consistent with the patterns observed in our cohort.

In addition to the beneficial effects of smoking cessation on health in general, it has a direct positive effect on HDL-C metabolism. At least partial recovery of smoking-induced impairments in HDL-C quantity and functionality by smoking cessation is evidenced by the fact that in addition to an increase in HDL-C concentration (mainly due to an increase in HDL-L particles)^36,86^, an improvement in CEC and antioxidant capacity is observed in individuals after smoking cessation^74,87^.

A major limitation of the present study is the relatively small sample size, which may result in limited statistical power and indicates that it would be useful to perform further analyses in a larger sample of different ethnicities to confirm our conclusions. Our analyses were adjusted for important relevant covariates; however, several environmental (such as air pollution), lifestyle (physical activity and diet), and individual factors (such as socioeconomic status, chronic diseases, medications, and their use or genetic background) that were not assessed in the present study may influence the HDL subfraction cholesterol profile and the effect of smoking. Furthermore, due to the cross-sectional nature of the present study, the results obtained can be considered as a point in time; therefore, no causal relationship between smoking and the HDL subfraction cholesterol profile can be inferred, but the results obtained can be taken into account in generating hypotheses about the relationship between them. Smoking and its exposure indicators (CPD, DoS, pack-years, and HSI) were assessed using self-reported data, and among nonsmokers, exposure to secondhand smoke cannot be excluded, which has the potential for recall bias or underreporting. Another limitation is that the present study did not include experiments on HDL function, which could have provided valuable insights into the significance of changes in HDL subfractions.

On the other hand, the present study has several strengths. This is the first study to examine the effects of smoking exposure on the cholesterol content of the ten HDL subfractions and three subclasses identified by the Lipoprint^®^ HDL system, extending previous comparisons between smokers and nonsmokers. An additional strength is that our analyses were adjusted for several factors affecting HDL-C levels and composition (for ethnicity, sex, age, BMI, systolic blood pressure, antihypertensive treatment, GOT level, diabetic status, self-reported financial status, and alcohol consumption).

## Conclusions

Our results confirm that smoking adversely affects the lipid profile and may contribute to an increased risk of atherosclerosis and other cardiovascular diseases. A quantitative decrease in total HDL-C levels and in the cholesterol content of HDL subfractions, mainly the HDL-I subclass and HDL-4 to HDL-7 subfractions, was observed in association with smoking and its severity. Further studies should investigate whether smoking-associated reductions in the identified HDL subfractions induce functional and biological alterations and whether these alterations contribute to the risk of developing cardiovascular diseases.

## Supplementary Information

Below is the link to the electronic supplementary material.

Supplementary Material 1

## Data Availability

Due to data protection and ethical concerns, the dataset(s) supporting the conclusions of this article are available upon request from the study coordinators, Prof. Róza Ádány (adany.roza@med.unideb.hu) and Dr. Péter Pikó (piko.peter@med.unideb.hu).

## Abbreviations

CPD: number of cigarettes smoked per day
DoS: duration of smoking
HSI: Heaviness of Smoking Index
CVDs: cardiovascular diseases
TC: total cholesterol
TG: triglycerides
LDL-C: low-density lipoprotein cholesterol
HDL-C: high-density lipoprotein cholesterol
LCAT: lecithin-cholesterol acyltransferase
CETP: cholesterol ester transfer protein
ApoAI: apolipoprotein AI
ApoB100: apolipoprotein B100
GPMSSP: General Practitioners’ Morbidity Sentinel Stations Program
HG: Hungarian general
GOT: glutamic-oxaloacetic transaminase
VLDL-C: very-low-density lipoprotein cholesterol
USPSTF: U.S. Preventive Services Task Force
BMI: body mass index
CEC: cholesterol efflux capacity
