# Supplementary material for: Dose‑dependent association of smoking exposure with HDL subfraction cholesterol content
Source: Sci Rep. 2026 Jun 11;16:23871. doi: 10.1038/s41598-026-57573-4 (PMC13434783; doi:10.1038/s41598-026-57573-4)
Supplement: Supplementary file 1 [file 41598_2026_57573_MOESM1_ESM.docx]

**Supplementary Table 1.** Association of smoking related indices with high-density lipoprotein (HDL) subfraction concentration (in %).

|  | Current smoker status | | | Number of cigarettes smoked per day | | Duration of smoking | | Pack year | | Heaviness of Smoking Index | |
| --- | --- | --- | --- | --- | --- | --- | --- | --- | --- | --- | --- |
|  | B (95% CI) | p-value^∞^ | B (95% CI) | | p-value^∞^ | B (95% CI) | p-value^∞^ | B (95% CI) | p-value^∞^ | B (95% CI) | p-value^∞^ |
| HDL-1 | 0.050 (-0.510 – 0.611) | 0.999 | 0.023 (-000.4 – 0.050) | | 0.490 | 0.005 (-0.019 – 0.028) | 0.999 | 0.011 (-0.009 – 0.032) | 0.939 | 0.124 (-0.038 – 0.285) | 0.665 |
| HDL-2 | -0.034 (-0.643 – 0.576) | 0.999 | 0.006 (-0.024 – 0.036) | | 0.782 | -0.003 (-0.028 – 0.023) | 0.928 | 0.004 (-0.018 – 0.027) | 0.991 | 0.065 (-0.111 – 0.242) | 0.777 |
| HDL-3 | -0.499 (-1.187 – 0.188) | 0.513 | -0.021 (-0.055 – 0.012) | | 0.525 | -0.013 (-0.042 – 0.016) | 0.945 | -0.009 (-0.034 – 0.016) | 0.980 | -0.087 (-0.287 – 0.112) | 0.975 |
| HDL-4 | -0.588 (-1.134 – -0.410) | 0.350 | -0.030 (-0.056 - -0.003) | | 0.310 | -0.022 (-0.045 – 0.002) | 0.690 | -0.017 (-0.037 – 0.003) | 0.994 | -0.129 (-0.288 – 0.030) | 0.999 |
| HDL-5 | 0.003 (-0.294 – 0.300) | 0.986 | -0.004 (-0.019 – 0.010) | | 0.786 | -0.001 (-0.014 – 0.011) | 0.849 | -0.001 (-0.012 – 0.009) | 0.913 | -0.003 (-0.089 – 0.083) | 0.999 |
| HDL-6 | 0.387 (-0.182 – 0.956) | 0.364 | 0.010 (-0.018 – 0.038) | | 0.827 | 0.009 (-0.015 – 0.033) | 0.888 | 0.006 (-0.015 – 0.027) | 0.987 | 0.044 (-0.120 – 0.209) | 0.892 |
| HDL-7 | 0.141 (-0.185 – 0.466) | 0.660 | 0.004 (-0.012 – 0.020) | | 0.834 | 0.004 (-0.010 – 0.018) | 0.990 | 0.002 (-0.010 – 0.014) | 0.915 | 0.003 (-0.091 – 0.098) | 0.947 |
| HDL-8 | 0.222 (-0.097 – 0.541) | 0.428 | 0.011 (-0.005 – 0.026) | | 0.577 | 0.009 (-0.004 – 0.023) | 0.613 | 0.007 (-0.004 – 0.019) | 0.999 | 0.044 (-0.049 – 0.136) | 0.999 |
| HDL-9 | 0.299 (0.004 – 0.594) | 0.235 | 0.008 (-0.006 – 0.023) | | 0.526 | 0.011 (-0.002 – 0.023) | 0.460 | 0.005 (-0.006 – 0.016) | 0.844 | 0.033 (-0.053 – 0.119) | 0.892 |
| HDL-10 | 0.357 (-0.705 – 1.419) | 0.727 | 0.004 (-0.048 – 0.056) | | 0.871 | 0.008 (-0.036 – 0.052) | 0.900 | -0.003 (-0.041 – 0.035) | 0.876 | -0.024 (-0.331 – 0.284) | 0.999 |
| Large HDL | -0.648 (-2.159 – 0.864) | 0.600 | 0.004 (-0.070 – 0.078) | | 0.919 | -0.017 (-0.080 – 0.047) | 0.604 | 0.003 (-0.052 – 0.058) | 0.999 | 0.088 (-0.349 – 0.525) | 0.999 |
| Intermediate HDL | -0.196 (-0.953 – 0.561) | 0.611 | -0.023 (-0.059 – 0.014) | | 0.687 | -0.011(-0.042 – 0.021) | 0.762 | -0.010 (-0.037 – 0.018) | 0.999 | -0.104 (-0.323 – 0.114) | 0.999 |
| Small HDL | 0.788 (-0.739 – 2.315) | 0.930 | 0.023 (-0.052 – 0.098) | | 0.816 | 0.026 (-0.037 – 0.089) | 0.999 | 0.008 (-0.047 – 0.063) | 0.901 | 0.047 (-0.395 – 0.490) | 0.834 |

^∞^p-values adjusted by the Benjamini‒Hochberg method. Multiple linear regression was adjusted for ethnicity, sex, age, BMI, systolic blood pressure, antihypertensive treatment, glutamic-oxaloacetic transaminase level, diabetic status, self-reported financial status, and alcohol consumption.
